# Healthcare Utilization of Patients With Acute Coronary Syndrome in Germany

**DOI:** 10.4021/cr279e

**Published:** 2013-07-11

**Authors:** Ariane Hoer, Susann Behrendt, Torsten Schmidt, Kathrin Lottmann

**Affiliations:** a IGES Institut GmbH, Friedrichstr. 180, D-10117 Berlin, Germany

**Keywords:** Acute coronary syndrome, Coronary heart disease, Delivery of health care, claims data, German

## Abstract

**Background:**

The aim of this study was to determine the health care utilization of patients with acute coronary syndrome (ACS) of one German statutory health insurance. The utilization of ambulatory services as well as of inpatient rehabilitation should be regarded. Moreover, the study should reveal the prescription of drugs for secondary prevention. Here, patients showing guideline corresponding prescriptions should be compared with patients without such prescriptions.

**Methods:**

A retrospective claims data analysis of one German statutory health insurance was conducted. Health care utilization was considered in the first year after an index hospitalization due to ACS. Beneficiaries for whom an ICD-10 discharge diagnosis of ACS was reported between January 1st 2007 and December 31st 2009 were included. In order to reveal differences in health care utilization depending on the type of ACS (STEMI versus NSTEMI/UA) stratified analyses were performed. Another stratification was done for patients with and without defined drug prescriptions.

**Results:**

From 45,188 patients with ACS almost three quarters were assigned to the group of NSTEMI/UA. For 8.9% of all ACS patients (18.74% STEMI, 8.89% NSTEMI/UA), inpatient post-hospital rehabilitation related to ACS was recorded. Ambulatory care related to CHD diagnosis was utilized by 77.6% of patients, more often by STEMI than by NSTEMI/UA patients. For 36.7% and 45.7% of ACS patients, a prescription of aspirin or clopidogrel was recorded, respectively, 79.4% of STEMI patients received at least one prescription for antiplatelet drugs, the corresponding proportion of NSTEMI/UA was 59.8%. A considerable part of patients without prescription dropped out within the first 90 days after the index event.

**Conclusions:**

A claims data analysis of one German statutory health insurance fund showed that health care utilization of ACS patients varied depending on the ACS type. It is necessary to distinguish between STEMI and NSTEMI/UA patients when discussing the ambulatory drug utilization.

## Introduction

Despite decreasing incidence, cardiovascular diseases (CVD) are still the leading cause of death in Germany and other industrialized countries. Among them, the most frequent causes of death are the ischemic heart diseases and the cardiovascular diseases. For 2010, official statistics show that on average 163 of 100,000 German residents died of ischemic heart diseases, thereof 72 of 100,000 residents died of myocardial infarction [1]. Furthermore, 148 of 100,000 women and 243 of 100,000 men had a hospital diagnosis of myocardial infarction in 2010 [2]. In addition to morbidity and mortality, coronary heart diseases (CHD) have an enormous impact on the costs of health care. According to the German Federal Statistical Office, 14.6% (37 billion Euros) of the total health care expenditures of 254 billion Euro in 2008 were spent on CVD. Thereof, CHD, including acute myocardial infarction, caused 2.4% (6.2 billion Euro) of total health care expenditures [3]. These high costs are mainly due to inpatient care including stent implantation and other surgeries [4].

CHD subsume the acute coronary syndrome (ACS) which describes a life-threatening condition of coronary heart disease including unstable angina pectoris, myocardial infarction, and sudden death [4, 5]. Patients with ACS are classified into three categories based on ECG changes and subsequent laboratory diagnostic: ST elevation myocardial infarction (STEMI), non-ST elevation myocardial infarction (NSTEMI), and unstable angina pectoris (UA) [5, 6].

Guidelines for the management of these ACS types are implemented [5, 6]: Emergency care, diagnosis and treatment in the acute phase have to be done inpatient. Post-hospital rehabilitation is recommended to STEMI patients and to NSTEMI patients with both, multiple modifiable risk factors and moderate to high risks [5, 6]. Due to the increased risk of secondary events and sudden death, the need of secondary prevention is evident [4, 7]. Apart from lifestyle changes, pharmacotherapy is recommended [5]: Aspirin as antiplatelet drug should be used first-line and has to be taken lifelong - either alone or for at least twelve months in combination with clopidogrel [5, 6]. For patients with stable angina, the use of β-blockers is recommended as first-line therapy [8]. Depending on co-morbidity, there is clinical evidence for the benefit of the usage of ACE inhibitors or statins [5, 6].

A recent German study on the drug-based secondary prevention of patients with myocardial infarction revealed a critical gap between evidence-based guidelines and recommendations and the health utilization reality. Post-hospital prescriptions of ASS resp. Clopidogrel were merely received by 66% resp. 61% of the study population. At least, 82% of the patients showed up ambulatory prescriptions β-blockers, but only 73% statins and 69% ACE inhibitors [9].

Along similar lines, further recent research with regard to the use of secondary prevention in patients with coronary heart disease (CHD) revealed that the usage resp. the patients’ compliance regarding these drugs is inappropriate. In high-income countries, only 64.1% of patients with CHD used antiplatelet drugs, 72.2% statins, 52.7% ACE inhibitators or angiotensin II receptor blocker (ARB), and 46.5% β-blockers. Drug usage (except β-blockers) significantly declined after the index event [7]. A German analysis evaluated that 47% of men and 59% of women received prescriptions for at least three of the four recommended drugs (antiplatelet drugs, β-blockers, ACE inhibitors, lipid-lowering agents) during the time between the initial myocardial infarction and a secondary event [10]. Another German study revealed that 83.6% of patients with a CHD history received antiplatelet drugs and 70.9% used β-blockers one year after the index event [11].

Referring to these estimations indicating a lack of secondary prevention on the one hand, and to the high burden of ACS on the other hand, we undertook a real life study based on claims data of one statutory health insurance (SHI) fund. The aim of this study was to determine the health care utilization of patients with ACS within the first year after hospitalization. In terms of drug therapy, the study was aimed not only at patients with drug prescriptions, but also at patients without prescriptions in order to contribute to a qualification of possible lack of health care.

Patients were stratified into two groups according to the type of ACS (STEMI and NSTEMI/UA) in order to identify potential health care differences. Results regarding drug prescriptions are discussed considering German and European ACS therapy guidelines [5, 6, 8].

## Methods

### Data and analysis population

The retrospective health care analysis was performed using claims data of one big statutory health insurance (SHI) fund from 2006 until 2009. This fund covers about 7 million SHI beneficiaries, corresponding to 10% of the German SHI population.

The analysis population included all beneficiaries of the SHI fund with at least one hospitalization due to ACS (discharge diagnosis) (Table 1) between January 1st 2007 and December 31st 2009. By setting a pre-period of one year (360 days) prior to the first recorded ACS event, the initial hospitalization due to ACS (index event) was identified. The follow-up period after the index event (discharge date) varies between beneficiaries; each person features its individual length of time after the ACS hospitalization, with a maximum of 2 years. Reasons for drop outs were death, end of insurance, and the end of the observation period.

**Table 1:** Discharge Diagnoses of ACS Considered for Selection of Analysis Population

ICD-10 (GM)	Title
I20.0	Unstable angina
I21.0	Acute transmural myocardial infarction of anterior wall
I21.1	Acute transmural myocardial infarction of inferior wall
I21.2	Acute transmural myocardial infarction of other sites
I21.3	Acute transmural myocardial infarction of unspecified site
I21.4	Acute subendocardial myocardial infarction
I21.9	Acute myocardial infarction, unspecified

For each patient of the analysis population the data set contained demographic information such as age and gender, inpatient and ambulatory diagnoses data (International Classification of Diseases, 10th Revision, German Modification) as well as data in respect to the utilization of inpatient and ambulatory health care as well as ambulatory drug prescriptions.

All statistical analyses were carried out using SAS (version 9.2).

### Index event and further cardiovascular events

Based on the diagnosis of the index event (Table 1), the analysis population was divided into two patient groups: 1), STEMI (ICD-10: I21.0; I21.1; I21.2; I21.3; I21.9); 2), NSTEMI/UA (ICD-10: I21.4; I20.0).

Moreover, the analysis focused the incidence or non-incidence of a secondary cardiovascular event within the individual observation period after the index event. A further cardiovascular event was defined by the diagnoses that were considered for determining the index ACS event (Table 1), as well as diagnoses of recurrent myocardial infarction (ICD-10: I22) and stroke (ICD-10: I63; I64).

Deaths could not be considered as secondary events because claims data do not provide any information on the cause of death.

### Post-hospital treatment: ambulatory medical services and inpatient rehabilitation

The utilization of medical services related to ACS was evaluated for the subsequent year (360 days) after discharge. For that, all ACS patients were included in the analysis except the ones who did not survive the index hospitalization. Ambulatory medical services were counted if the related treatment case was recorded together with a diagnosis considered for identification of CHD (ICD 10: I20-I25). We presumed that after the patient was treated in hospital due to ACS - which is an acute condition - the subsequent ambulatory care will be done for the underlying chronic disease, which is CHD. In order to validate this assumption, the ambulatory cases which were recorded with diagnoses considered as further ACS events were counted as well. It has to be noted that, in German healthcare system, one ambulatory treatment case contains all claimed ambulatory services of the same physician within one quarter. Therefore, neither the number of ambulatory treatment cases nor the number of claimed services is equal to the number of ambulatory visits.

Inpatient rehabilitation cases were included, if the corresponding primary diagnosis conformed to the diagnoses characterizing a recurrent ACS event. Data on ambulatory post-hospital rehabilitation were not available.

Frequency distributions, arithmetic means and standard deviation were used as descriptive statistical measures in order to illustrate the utilization of in- and ambulatory care in patients with ACS in Germany.

### Ambulatory medication

On the basis of the ambulatory prescription data, the analysis investigated the pharmacotherapy within the first year after the index event. One focus was on the antiplatelet drugs aspirin and clopidogrel. Those prescriptions were included, if they occurred within the subsequent one hundred days after the ACS index event (so-called index prescription).

The second focus of the analysis was on other drug therapy with regard to secondary prevention after ACS. The following drug classes were included: statins, β-blocker and agents with effects on RAS (ACE inhibitors, ARBs, and fixed combos of these drugs with other compounds). Drug therapy was considered if at least one corresponding prescription per patient had been documented within the first year after the index event.

For both, antiplatelet and other drugs for secondary prevention after ACS, the number of patients with this medication was displayed per drug class. Analysis of prescriptions was stratified to STEMI and NSTEMI/UA patients as well as patients with and without a secondary cardiovascular event. With regard to antiplatelet drugs, patients for whom no index prescription was found, were characterized more detailed by variables who may lead to non-prescription: mainly age (as general indicator for possible contraindications), sex (which might have influenced prescription), death within the first 90 days after the index event (which would have prevented prescription), ulcer diagnoses (ICD 10: K25-K28; indicating a contraindication for antiplatelet drugs), prescriptions of vitamin K-antagonists (which are usually not prescribed together with antiplatelet drugs) and antiplatelet drug prescription before index event (which might indicate sufficient supply with antiplatelet drugs) were considered.

With regard to other drugs for secondary prevention, patients without any prescription as well as the group of patients without statin prescriptions within the first 180 days after the index event were characterized as well. This sub-analysis focused the variables age, sex, prescriptions for defined other drugs, and the drop out rates. Frequency distributions, arithmetic means and standard deviances were calculated.

## Results

### Study population

The analysis population consisted of 45,188 persons who had at least one inpatient ACS event defined as an index event within the period between January 1st 2007 and December 31st 2009 and who survived the first ACS hospitalization. According to the discharge diagnoses of the index event, almost three quarters of the population belonged to the group of NSTEMI/UA (74.71%). Table 2 summarizes the baseline characteristics of the population.

**Table 2:** Baseline Characteristics of the Study Population

	STEMI	NSTEMI/UA	Total
Patients (n)	11,430	33,758	45,188
Women (%)	42.89	49.56	47.87
Age (%)			
0 – 50	11.68	6.27	7.64
51 – 60	18.83	13.78	14.92
61 – 70	26.06	26.61	26.47
71 – 80	25.32	30.87	29.47
81 +	18.64	22.46	21.50

Most of the patients could not be observed for two years because they dropped out earlier: 29.6% (13,383) of the patients dropped out within the first year after the index event, and after two years the drop-out summed up to 57.3% (25,911) of the analysis population.

**Figure 1:**
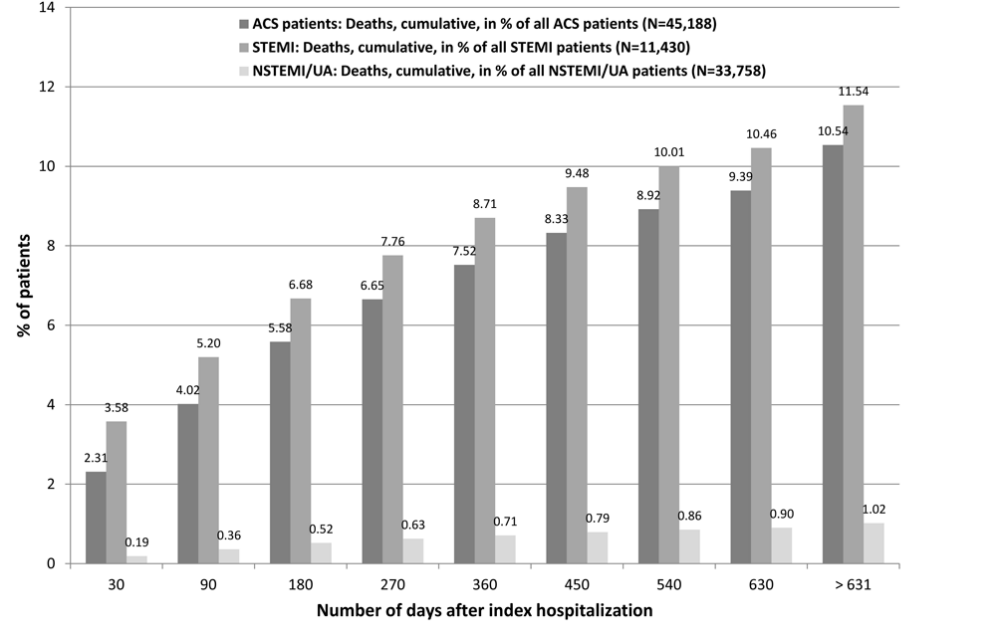
Drop out of ACS patients due to deaths after discharge from the index hospitalization.

Within the first 30 days after discharge, 1,045 patients (2.31% of all ACS patients) died, the cause of death is not part of the claim data set. Further 1,183 patients (2.62% of all ACS patients) dropped out because their index event occurred close to the end of the observation period (December 31st 2009). For 57 patients (0.13% of all ACS patients) other reasons lead to drop out. Figure 1 shows the increase of the proportion of drop outs due to deaths from discharge date of the index event until the end of the data set observation period (December 31st 2009).

Again, it is important to note that no information on the cause of death was available. Thus, the results of Figure 1 refer to the mortality of ACS patients (not to lethality). Comparing the ACS patient group death rates, it is clearly recognizable that the drop out of STEMI patients due to death is much higher than the one of NSTEMI/UA patients. Already within the first 30 days after the index hospitalization, 3.58% of STEMI patients (vs. 0.19% of NSTEMI/UA patients) died. After one year 7.52% of ACS patients dropped out due to death (8.71% patients of STEMI; 0.71% patients of NSTEMI/UA).

### Secondary cardiovascular event and deaths

For more than one tenth of the analysis population (5,304 patients) at least one further cardivascular event after the index event was observed. This proportion was similar with regard to patient groups: 11.08% (1,349 patients) of STEMI patients, 11.72% (3,955 patients) of NSTEMI/UA patients. Four fifth of these ACS patients (81.15%) experienced the further cardiovascular event within the first year after the index event. This applies to 84.80% of STEMI patients with further event and 79.90% of NSTEMI/UA patients with further event. In higher age groups, secondary cardiovascular events occurred more frequently than in younger patients.

### Post-hospital treatment: ambulatory medical services and rehabilitation

The frequency of ACS-related ambulatory medical services within the first year after the index event was low, as it has been expected. For only one third of all ACS patients the analysis could identify at least one outpatient visit related to an ACS diagnosis. In contrast, the results reveal much higher frequencies of CHD-related utilization of outpatient care (Table 3). Whereas more than four fifth of the analyzed STEMI population (84.61% of STEMI patients) visited an office-based physician, this applied to three quarter of NSTEMI/UA patients (75.75%).

**Table 3:** Frequency of Ambulatory Medical Care Within the First 360 Days After the Index Event in Comparison: ACS Diagnosis Versus CHD Diagnosis

	STEMI (N = 11,430)	NSTEMI/UA (N = 33,758)	Total (N = 45,188)
ACS			
Patients with ACS-related ambulatory case (n (%))	7,233 (63.28)	8,360 (24.76)	15,593 (34.51)
CHD			
Patients with CHD-related ambulatory case (n (%))	9,671 (84.61)	25,404 (75.25)	35,075 (77.62)
CHD-related ambulatory cases per patient (mean, SD)	8.87 ± 6.00	6.85 ± 5.36	7.41 ± 5.61

Thus, in respect to the CHD-related ambulatory care, the frequency of ambulatory treatment cases varied only low comparing the STEMI and NSTEMI/UA patient groups. According to the present data for each ACS patient a mean of 7.41 ± 5.61 ambulatory visits referring to CHD diagnosis was observed within the first year after the index hospitalization. The mean number of recorded treatment cases for STEMI patients was over-average (8.87 ± 6.00).

Furthermore, the analysis regarded the utilization of post-hospital inpatient rehabilitation due to ACS. For 4,018 patients (8.89%) on average one inpatient rehabilitation stay was reported within the first year after the index event. Only for 5.56% of the NSTEMI/UA this health service was observed; in contrast, the corresponding proportion for STEMI patients was 3.37-fold as high as the one of NSTEMI/UA patients (18.74%).

### Ambulatory prescriptions

For about two thirds of all ACS patients an index prescription of at least one of the considered antiplatelet drugs aspirin, clopidogrel, prasugrel (approval in Germany 2009) and fixed combos was reported for the first 100 days after the index event. Prasugrel and fixed combinations did not show high measures (0.43% and 0.17% of all ACS patients), but clopidogrel was found to be the antiplatelet drug prescribed to the largest proportion of patients (45.73%), followed by aspirin (36.70%). In comparison to NSTEMI/UA patients the proportion of STEMI patients with clopidogrel prescriptions was substantially higher (65.25% vs. 39.12%), similar results were revealed for aspirin (49.56% vs. 32.35%).

By implication, for about one third of the ACS patients (35.19%) an index prescription was not detectable. The corresponding proportion for NSTEMI/UA was 40.16% and was twice as high as for STEMI patients (20.53%). A more detailed analysis compared the ACS patients with resp. without an index prescription of antiplatelet drugs. Table 4 displays the key variables which were included in order to characterize the patients without index prescriptions.

**Table 4:** Characteristics of ACS Patients With and Without an Index Prescription of Antiplatelet Drugs

	With index prescription for antiplatelet drug			No index prescription for antiplatelet drug		
	NSTEMI/UA	STEMI	Total	NSTEMI/UA	STEMI	Total
Patients (n (%))	20,202 (100.00)	9,083 (100.00)	29,285 (100.00)	13,556 (100.00)	2,347 (100.00)	15,903 (100.00)
Age (Means ± SD)	71.22 ± 11.32	67.14 ± 12.98	69.95 ± 12.01	69.63 ± 13.12	69.58 ± 14.57	69.62 ± 13.35
Patients older than 75 years (n (%))	7,683 (38.03)	2,533 (27.89)	10,216 (34.88)	4,708 (34.73)	880 (37.49)	5,588 (35.14)
Women (n, %)	9,117 (45.13)	3,694 (40.67)	12,811 (43.75)	7,614 (56.17)	1,208 (51.47)	8,822 (55.47)
Patients with ulcer diagnosis within the year before index event (ICD-10: K25-K28) (n (%))	624 (3.09)	190 (2.09)	814 (2.78)	425 (3.14)	54 (2.30)	479 (3.01)
Patients with prescription of antiplatelet drugs within the last 3 months before index event (n (%))	5,042 (24.96)	8,35 (9.19)	5,877 (20.07)	1,690 (12.47)	2,13 (9.08)	1,903 (11.97)
Patients with prescription of vitamin-k-antagonists within the first 100 days after index event (n (%))	962 (4.76)	448 (4.93)	1,410 (4.81)	1,610 (11.88)	293 (12.48)	1,903 (11.97)
Drop out within the first 30 days after the index event (n (%))	511 (2.53)	207 (3.38)	818 (2.79)	1,038 (7.66)	429 (18.28)	1,467 (9.22)
Drop out within the first 90 days after the index event (n (%))	1,787 (8.85)	788 (8.68)	2,575 (8.79)	2,254 (16.63)	705 (30.04)	2,959 (18.61)

First of all, it clearly strikes that nearly one fifth (18.61%) of the patients without prescriptions dropped out within the first three months after the index event. The high proportion of STEMI patients without an index prescription may refer to the fact that 30.09% were not included in the analysis for longer than three months. Concerning age and sex, slight differences became apparent: While the average age level is similar for both, the proportion of STEMI patients older than 75 years within the group without a prescription is over-average and 10% higher than the proportion for STEMI patients of the group with an index prescription (37.49% vs. 27.89%). Besides, it strikes that patients without an index prescription more often were female than male in comparison to the other group. Another variable considered was the presence of an ulcer-related diagnosis before the index event happened. Observed differences were marginal for this variable: on average, for only 3% of the ACS patients with as well as without an index prescription an ulcer diagnosis was observed. The absence of an index prescription could also refer to sufficient drug supply due to prescription before the index event and used up after the index event by the patient. As our analysis shows, this might apply to 11.97% of the patients without an index prescription. In contrast, one fifth of the other group had previous antiplatelet drug prescription. More than one tenth (11.97%) of the patients without an index prescription showed at least one prescription of vitamin K-antagonists within the first one hundred days after the index event. In contrast, this applied to only 4.81% of patients with an index prescription of the considered antiplatelet drugs. The proportion of NSTEMI/UA patients without an index prescription but supplied with vitamin k-antagonists was nearly as high as the corresponding proportion of STEMI patients (11.88% vs. 12.48%).

**Figure 2:**
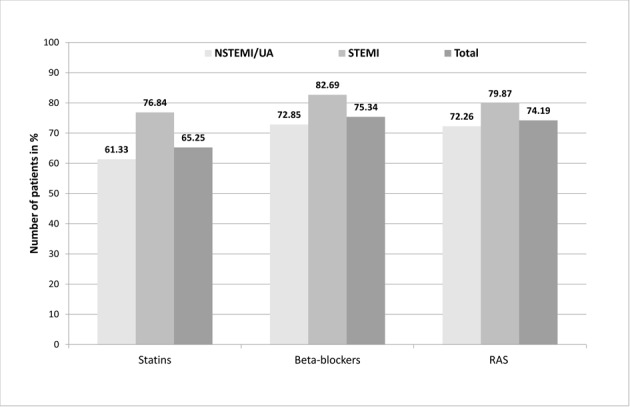
ACS patients with at least one prescription of the considered drugs for secondary prevention after ACS.

In addition, our analysis regarded prescriptions of statins, β-blocker and drugs acting on the RAS as other drugs for secondary prevention which have been prescribed within the first year after the index event as well. For 89.28% of all ACS patients, the analysis displayed at least one prescription for statins, β-blocker and RAS agents within the first 180 days (STEMI: 91.21%; NSTEMI/UA: 88.63%). The distribution for each drug class is shown in Figure 2.

For all drugs the analysis revealed high prescription rates: β-blockers were the dominant drug class followed by drugs acting on the RAS. Both were prescribed to about three quarters of the ACS patients in total, however, only two thirds of NSTEMI/UA patients received drug prescriptions within the first six months after the index event. STEMI patients showed the highest proportions of prescriptions for all drugs. Especially in the case of statins there was a large difference in prescription rates with 15.51% between STEMI and NSTEMI/UA.

The remaining 10.72% of ACS patients who did not show any prescription of the drugs considered are characterized in Table 5. Most of these patients (71.13%) dropped out within the first three months. While 35% of alls ACS patients already dropped out within 30 days after the index event the proportion of STEMI patients was much higher (50.55%).

**Table 5:** Characteristics of ACS Patients Without Any Prescription of Statins, β-Blocker and RAS Agents Within the First 180 Days After the Index Event

	NSTEMI/UA	STEMI	Total
Patients (n (%))	3,837 (100.00)	1,005 (100.00)	4,842 (100.00)
Age (mean ± SD)	67.32 ± 15.72	69.07 ± 16.31	67.68 ± 15.85
Patients older than 75 years (n (%))	1,302 (33.93)	398 (36.60)	1,700 (35.11)
Women (n (%))	2,017 (52.57)	517 (51.44)	2,534 (52.33)
Patients with index prescription of antiplatelet drugs (n (%))	558 (14.54)	121 (12.04)	679 (14.02)
Drop out within the first 30 days after the index event (n (%))	1,199 (31.25)	508 (50.55)	1,707 (35.25)
Drop out within the first 90 days after the index event (n (%))	2,595 (67.63)	849 (84.48)	3,444 (71.13)

Due to the low prescription rates for statins, our analysis regarded patients without statin prescriptions separately (n = 15,702). Table 6 summarizes the results. First of all, it can be noted that much more patients without a statin prescription dropped out within the first 30 days (10.87%) resp. 90 days (21.93%) after the index event than patients with statin prescriptions (1.96% resp. 7.09%). Among patients without statin prescriptions the proportion of patients with prescription for β-blockers and drugs acting on the RAS was only 53.39% and 54.08%, respectively. In contrast, for patients who showed at least one prescription of statins within the respective time period, the proportion with prescription for β-blockers and drugs acting on RAS was 1.6-fold higher (87.03% vs. 53.39% and 84.89% vs. 54.08%, respectively). There were results revealing only small differences between STEMI and NSTEMI/UA patients cocerning these characteristics. Besides, only one third (33.08%) of the patients without statins had an index prescription of the considered antiplatelet drugs. The corresponding proportion for STEMI patients was substantially higher than for NSTEMI/UA patients. Looking at the patients with prescribed statins, this number was more than twice as high (76.63%). Comparing the proportion of patients older than 75 years it was found that the patients, for whom no statin prescription was observed, were older than those with statin prescriptions.

**Table 6:** Characteristics of ACS Patients Without a Prescription of Statins Within the First 180 Days After the Index Event

	Statins prescription			No statins prescription		
	NSTEMI/UA	STEMI	Total	NSTEMI/UA	STEMI	Total
Patients (n (%))	20,703 (100.00%)	8,783 (100.00%)	29,486 (100.00%)	13,055 (100.00%)	2,647 (100.00%)	15,702 (100.00%)
Age (mean ± SD)	69.96 ± 10.84	66.20 ± 12.53	68.85 ± 11.49	71.57 ± 13.81	72.34 ± 14.87	71.70 ± 14.00
Patients older than 75 years (n (%))	6,760 (32.65)	2,175 (24.76)	8,935 (30.30)	5,631 (43.13)	1,238 (46.77)	6,869 (43.75)
Women (n (%))	9,213 (44.50)	3,459 (39.38)	12,672 (42.98)	7,518 (57.59)	1,443 (54.51)	8,961 (57.07)
Patients with index prescription of antiplatelet drugs (n (%))	15,282 (73.82)	7,902 (89.97)	23,184 (78.63)	4,920 (37.69)	1,179 (44.54)	6,099 (38.84)
Patients with prescription of β-blockers within 180 days after index event (n (%))	17,552 (84.78)	8,110 (92.34)	25,662 (87.03)	7,041 (53.93)	1,342 (50.70)	8,383 (53.39)
Patients with prescription acting on RAS within 180 days after index event (n (%))	17,168 (82.93)	7,863 (89.53)	25,031 (84.89)	7,226 (55.35)	1,266 (47.83)	8,492 (54.08)
Drop out within the first 30 days after the index event (n (%))	350 (1.69)	228 (2.60)	578 (1.96)	1,199 (9.18)	508 (19.19)	1,707 (10.87)
Drop out within the first 90 days after the index event (n (%))	1,446 (6.98)	644 (7.33)	2,090 (7.03)	2,595 (19.88)	849 (32.07)	3,444 (21.93)

## Discussion

The present study fills a significant lack of claims data analyses focusing on ACS and corresponding health care as it is the first retrospective cohort study targeting not only health care utilization but also the non-utilization in Germany. The analysis included more than 45,000 patients with ACS with a follow-up of up to 2 years after the first ACS event.

The aim of this study was to map the health care utilization of patients with ACS. Primary focus applied on the one hand to the utilization of ambulatory and inpatient health care; the latter included inpatient post-hospital rehabilitation as well. On the other hand, ambulatory drug prescriptions (antiplatelet drugs; other drugs for secondary prevention) were a central part of the analysis. For this purpose, the analysis was stratified to STEMI and NSTEMI/UA patients.

Summarizing the key results, it should be discussed how these results fit to other relevant studies as well as to the recommendations of current guidelines for ACS management. First of all, one result of our study underlines the importance of secondary prevention for ACS patients within the first year after the index hospitalization: on four fifth of patients affected by another cardiovascular event, the second event occurred within 360 days after the first ACS event.

Rehabilitation is recommended after STEMI and for defined cases of NSTEMI [5, 6]. For only 8.89% of the analysis population of our study (18.74% of STEMI and only 5.56% of NSTEMI/UA patients), an inpatient rehabilitation following the index event was reported. Another German study revealed that 10% of patients suffering a myocardial infarction and with a strong indication for inpatient rehabilitation received any rehabilitation [4]. According to the European guideline of the management of acute myocardial infarction in patients presenting with persistent ST-segment elevation, there is probably no difference between home- and hospital-based rehabilitation [6]. The claims data shows only a part of the rehabilitation measures due to ACS: data do not provide information on inpatient post hospital rehabilitation at expense of pension funds. This is important to note, because all those rehabilitation measures which shall restore the capacity to work are not reimbursed by the SHI funds. Data on ambulatory rehabilitation were available neither. This may lead to an underestimation of post-hospital rehabilitation frequency. In conclusion, an assessment, if rehabilitative care in ACS patients is sufficient, is not possible.

With regard to ambulatory health care service, there were no clear indications of under usage. In total, about 78% of the patients demanded at least one ambulatory service within the first year after the index hospitalization. Among the STEMI patients this proportion was nearly 85%. Considering that there was some drop out due to death (about 5% of STEMI patients during the first 30 days after discharge from the index hospitalization), it can be assumed that the usage of ambulatory health care service is quite good at least in this patient group. Nevertheless, regarding the NSTEMI patients further investigations should clarify potential correlation of ambulatory visits and low frequency of ACS-related prescriptions.

According to clinical guidelines concerning ACS-related secondary prevention, aspirin should be taken lifelong [5, 6]. In contrast to this recommendation, the analysis showed that more than one third of the ACS patients (35.19%) did not receive prescriptions of the considered antiplatelet drugs aspirin, clopidogrel, prasugrel and corresponding fixed combinations.

With regard to aspirin, the rate is even lower: 36.70% of the patients showed at least one aspirin prescription within the first hundred days after the index event. The results revealed furthermore that clopidogrel was the dominant agent for both STEMI and NSTEMI/UA although the usage of clopidogrel was substantially higher for STEMI than for NSTEMI/UA patients. It is important to note that the results of our study may underestimate aspirin prescription prevalence due to the fact that since 2004 aspirin is not fully reimbursed. Therefore, only a proportion of unknown quantity of aspirin prescriptions is covered by the claims data. The extent of this potential underestimation cannot be determined. However, our results strongly indicate some underusage of these antiplatelet agents after ACS.

The extent of an underusage varies between the patient groups. Thus, only 16% of STEMI patients did not show any index prescription, more than four fifth of them did which is a quite good value, considering that there were some drop outs and that there may be some underestimation of aspirin prescriptions. In contrast, NSTEMI/UA patients had fewer prescriptions of antiplatelet drugs. This result emphasizes the necessity to distinguish between these two patient groups when evaluating the deficits of care.

A recent German study on secondary prevention after myocardial infarction came to the conclusion that there is a substantial gap between health care reality and clinical guidelines. The rates of patients with at least one prescription of aspirin resp. clopidogrel amounted to 66% resp. 61% of the study population [9]. Considering the decreasing prescription prevalence over time reported by this study, an alarming health care situation of ACS patients could be assumed. Yusuf et al (2011) also reported similar results of insufficient antiplatelet drug therapy. The study concluded that, in high income countries (not including Germany), only 64.1% of patients with CHD received antiplatelet drugs [7]. A German registry showed higher proportions: in 2009/2010, about 81% of men and 76% of women with myocardial infarction received antiplatelet drugs before being affected by another infarction [12]. Publications of both studies separately show neither aspirin nor clopidogrel. Thus, recent studies indicate that there may be a significant proportion of ACS patients who may not benefit from an antiplatelet drug therapy.

Studies hardly focus on those patients who did not receive prescriptions for antiplatelet drugs. In the frame of our study, we undertook further analyses in order to describe those patients concerning selected variables. Even though we did not adjust the results by contraindications in general, we compared the frequency of ulcer-related diagnoses between the patients with vs. patients without prescriptions of antiplatelet drugs. Estimating a diagnosis prevalence of 3% for both groups, one might assume that the presence of ulcer is not a dominant factor for the absence of the relevant prescriptions. A more probable explanation for the high proportion of patients without prescriptions is the drop out due to mortality and other reasons within the first three months after the index event. A relevant number of the patients who dropped out early (18.61%) simply did not have an occasion to get an ambulatory prescription of antiplatelet drugs. STEMI patients with a lack of prescriptions of these drugs were substantially older than STEMI patients who received such prescriptions, which may be an indicator for some unspecific risk leading to the reluctance in prescribing antiplatelet drugs. This relation could not be affirmed for NSTEMI/UA patients. The descriptive results concerning previous drug prescriptions before the index event could not reveal a plausible explanation for non-prescription of antiplatelet drugs after the index event. Thus, there is a substantial need of further studies which (a) focus not only on the patients with relevant drug prescriptions but also on the ones without these prescriptions; (b) identify potential variables influencing the patient-related reasons for non utilization patterns; and (c) calculate the impact of the influencing factors on the health care reality, here on drug therapy.

In addition to antiplatelet drugs, clinical guidelines for the management of ACS recommend the lifelong use of β-blockers in the absence of contraindications. Depending on co-morbidity, there is clinical evidence for the use of RAS agents and statins. The results of our studies cannot be distinctly interpreted but they clarify that the vast majority of the analysis population had at least one prescription of the considered agents. The prescription rates of statins covering less than two thirds of the patients with at least one prescription were rather low compared to β-blockers and drugs acting on the RAS. A more detailed view on these patients revealed that a significant part of these patients received β-blockers and/or RAS agents and/or antiplatelet drugs, but these proportions were much lower than for patients with statin prescriptions. Thus, there may be different reasons for non-prescription: higher risks for adverse events as well as favorable risk profiles concerning cardiovascular risk or as well as the patients' refusal to take drugs or visit a doctor.

Other studies reported heterogeneous prescription prevalence for these drug groups. Basically, the proportion of ACS patients using statins is varying between two thirds and three quarter [7, 12]. These figures correspond to the present study. A recent German study on drug therapy after myocardial infarction revealed that 82% of the patients received β-blockers, 73% statins and 69% ACE inhibitors [9].

Our analysis was based on SHI claims data which are routinely generated independently of scientific objectives and interests. Even though the dataset allowed the analysis of more than 45,000 patients with ACS, it cannot be assumed that the results are representative for all ACS patients in Germany or at least for the SHI beneficiaries. Potential clientele effects due to the dataset of just one SHI fund may exist.

### Conclusion

This retrospective claims data analysis allowed the mapping of ACS patient-related health utilization patterns over two years after the first ACS event by using ambulatory diagnoses and services data, inpatient data including inpatient rehabilitation as well as information on ambulatory prescriptions for secondary prevention. The analysis revealed that about 80% of the secondary cardiovascular events occurred within the first 360 days after their first ACS event. This emphasizes the need of adequate secondary prevention and care while the data showed a lack of secondary drug prescriptions. Further, the results suggest differentiating between STEMI and NSTEMI/UA patients when discussing (reasons for) health care utilization as well as non-utilization.

In order to assess whether a medical treatment is in accordance to current ACS management guidelines or not, clinical parameter, information about the patient history as well as further information concerning risk factors resp. health behavior are needed. They are not part of SHI claims data used for our analysis. Embedded into a SHI claims data analysis, more detailed investigations should be undertaken considering coronary interventions, ACS related comorbidity and diagnoses related to contraindications of antiplatelet drugs and other drug therapy for secondary prevention as well as a follow-up of more than one year. Patients without prescriptions or other usage of health services should be a central part in order to identify not just gaps but to qualify statements of insufficient health care and last but not least to optimize provision of health care.
